# Urban-Hazard Risk Analysis: Mapping of Heat-Related Risks in the Elderly in Major Italian Cities

**DOI:** 10.1371/journal.pone.0127277

**Published:** 2015-05-18

**Authors:** Marco Morabito, Alfonso Crisci, Beniamino Gioli, Giovanni Gualtieri, Piero Toscano, Valentina Di Stefano, Simone Orlandini, Gian Franco Gensini

**Affiliations:** 1 Institute of Biometeorology, National Research Council, Florence, Italy; 2 Interdepartmental Centre of Bioclimatology, University of Florence, Florence, Italy; 3 Fondazione per il Clima e la Sostenibilità, Florence, Italy; 4 Department of Agrifood Production and Environmental Sciences, University of Florence, Florence, Italy; 5 Clinica Medica e Cardiologia, University of Florence, Florence, Italy; Örebro University, SWEDEN

## Abstract

**Background:**

Short-term impacts of high temperatures on the elderly are well known. Even though Italy has the highest proportion of elderly citizens in Europe, there is a lack of information on spatial heat-related elderly risks.

**Objectives:**

Development of high-resolution, heat-related urban risk maps regarding the elderly population (≥65).

**Methods:**

A long time-series (2001–2013) of remote sensing MODIS data, averaged over the summer period for eleven major Italian cities, were downscaled to obtain high spatial resolution (100 m) daytime and night-time land surface temperatures (LST). LST was estimated pixel-wise by applying two statistical model approaches: 1) the Linear Regression Model (LRM); 2) the Generalized Additive Model (GAM). Total and elderly population density data were extracted from the Joint Research Centre population grid (100 m) from the 2001 census (Eurostat source), and processed together using “Crichton’s Risk Triangle” hazard-risk methodology for obtaining a Heat-related Elderly Risk Index (HERI).

**Results:**

The GAM procedure allowed for improved daytime and night-time LST estimations compared to the LRM approach. High-resolution maps of daytime and night-time HERI levels were developed for inland and coastal cities. Urban areas with the hazardous HERI level (very high risk) were not necessarily characterized by the highest temperatures. The hazardous HERI level was generally localized to encompass the city-centre in inland cities and the inner area in coastal cities. The two most dangerous HERI levels were greater in the coastal rather than inland cities.

**Conclusions:**

This study shows the great potential of combining geospatial technologies and spatial demographic characteristics within a simple and flexible framework in order to provide high-resolution urban mapping of daytime and night-time HERI. In this way, potential areas for intervention are immediately identified with up-to-street level details. This information could support public health operators and facilitate coordination for heat-related emergencies.

## Introduction

Short-term impacts of high air temperature on human health are well known [1, 2]. Furthermore, different city-specific temperature thresholds have been assessed worldwide [3].

High temperature has the worst impact on the elderly [4]. The highest vulnerability of the elderly to heat is related to physiological, health and socio-economical status. The key pathophysiological problem is early dehydration due to age-related reduced thirst and the capacity to conserve salt and water [5]. Furthermore, thirst might also be suppressed by the consumption of certain medications used more frequently in elderly subjects due to a relatively higher percentage of illness and disability [5]. The need to use medications is often related to an impaired thermoregulatory function and a consequently diminished or delayed physiological ability to maintain core temperature within safe and acceptable limits. The worsening of other factors such as living conditions, family and/or social support and the ability to access medical-care systems might represent additional risk factors.

People living in heavily built-up areas, especially if they live in old buildings without insulation or air conditioning, are at a greatest risk [1] because of the urban heat island (UHI) effect. This latter phenomenon is characterized by a metropolitan area significantly warmer than its surrounding suburban or rural areas due to increased thermal storage capacity during the day and released night-time radiation [6]. The UHI is one of the most significant human-induced changes to the earth's surface and local climate [7], also with effects on human health [8].

It is well known that temperature has a great spatial variability in metropolitan areas because of the complex interaction of physical variables, such as surface and air temperature gradients, turbulent transport, and surface energy exchange.

Temperatures typically rise in the most densely urbanized areas, creating a concomitance of adverse environmental conditions and high levels of exposure of the population that need to be assessed by integrated methodologies for accurately resolving spatial scales.

As meteorological station networks are generally scattered and often located in suburban areas (i.e. airports or parks), they only provide partial representations of air-temperature variations in heterogeneous urban/suburban environments. For this reason, tools for intra-urban hazard risk assessments are required. In particular, the development of geoscience and the wide availability of remote sensing data allow for ongoing accurate estimations of the Land Surface Temperature (LST) and the Normalized Difference Vegetative Index (NDVI). LST-NDVI relationships have already been studied through linear regression analyses over a wide range of moisture and climatic/radiation regimes [9] and specifically in urban environments [10]. However, non-linear relationships between LST and NDVI might also occur [11] and additional methods accounting for the non-linearity of the relationships should be considered.

Remote sensing data, together with spatial socio-demographic datasets describing the exposed vulnerable population, are valuable indicators of urban hazard health risk analyses. We hypothesize that these datasets, if available at high spatial resolution, might represent an essential contribution to prevent the heat impact on human health with detailed information at the urban small-area level.

Several examples of heat risk assessment in urban areas exist in the USA [12], Canada [13], UK [14] and France [15]. Despite the fact that Italy has one of the highest median ages in the world with the greatest proportion of elderly people in Europe (second in the world to Japan) [16], there is a lack of information concerning intra-city spatial heat-related health risks. In Italy, remote sensing data applications are only available for investigating the UHI in a few cities [17]. Increasingly, policy makers require detailed information on the exposed population, in order to know the location of vulnerable groups and have a synthetic risk indicator related to extreme heat events.

Spatial population density is the main socio-demographic indicator of heat exposure generally used in risk assessment studies. Population and housing censuses have a long tradition in the European Union. However, the way the census methods are developed in each country depends on the availability of data sources and technology, data protection requirements, the burden on the respondents, and the census operation costs. More recently, the Joint Research Centre (JRC) has produced a raster dataset representing the 1-km^2^ European population density on a 100-m grid by using the downscaling method described in Gallego [18]. To our knowledge, this demographic dataset has never been used for spatial heat-related health risk studies.

The main aim of this study was to develop high-resolution daytime and night-time heat-related population risk maps, specifically referring to the elderly (people aged 65 or over) and to summer (May-September), for the major Italian cities with different geographical features (coastal and inland at different latitudes). In order to achieve the main aim, a preliminary analysis was addressed to define a reliable downscaling procedure (100 m) in order to obtain a long time-series (2001–2013) of MODIS LST data. For each city, Heat-related Elderly Risk Index (HERI) maps were developed through the interaction of the natural hazard layer (LST) with spatial demographic data by using “Crichton’s Risk Triangle” [19] framework.

## Materials and Methods

### Period and study-areas

This study was carried out during the warmest period of the year (May-September) over a 13-year period (2001–2013). The city selection criterion was based on the number of residents. Eleven major Italian cities with more than 200,000 inhabitants were selected from all over the country: five in the North (Milan, Padua, Turin, Bologna and Genoa), two in the Centre (Florence and Rome), and four in the South (Bari, Naples, Palermo and Catania) (S1 Fig).

These cities are located in different geographical (S1 Table) and climatic conditions. Milan, Padua, Bologna and Florence are inland plain cities. Turin is a hybrid inland plane/hill city (average altitude of 239 m a.s.l). Rome is considered a hybrid coastal/inland city because of the relatively long distance between the city-centre and the seaside (more than 20 km). For this reason, the results for the city of Rome are shown and discussed together with the inland cities. Genoa, Bari, Naples, Palermo and Catania are coastal plain cites. According to the Köppen climate classification, Milan, Padua, Turin and Bologna are characterized by a humid, subtropical climate, with little or no influence from the sea, and moderately hot summers. Genoa and Florence have a borderline humid subtropical and Mediterranean climate, with a strong (Genoa) and moderate (Florence) influence from the sea and hot-humid summers. The other cities are characterized by a typical Mediterranean climate, with significant influence from the sea and hot-dry summers.

The borders delimiting each city study-area were defined in order to include, besides the main city itself, an outer belt. The latter was represented by the municipalities of all satellite residential towns, suburbs or settlements around the city.

### Study design: spatial heat-related elderly risk assessment

The study design focuses on “Crichton’s Risk Triangle” hazard-risk assessment methodology [19] adopted through the ASCCUE (Adaptation Strategies for Climate Change in the Urban Environment) project [20]. The ASCCUE project aimed to improve understanding of the consequences of climate change for urban areas and testing of appropriate adaptation responses through assessment of impacts by using concepts of risk. In this study, the risk concept is represented by harmful human health consequences for elderly people resulting from the interaction between three components that form a triangle: natural hazard, exposure, and vulnerability. The risk is defined as a function of these three components. If any component or “side” of the triangle is zero, then there is no risk. The natural hazard is defined as the LST increase. Exposure to the natural hazard is depicted by the total population census data, the vulnerability by the elderly population (over 65). The final risk map is generated from the spatial interaction of all three components.

### Hazard layer: daytime and night-time summer LST layers

The hazard layer is obtained for the period 2001–2013 using two remote sensing MODIS data products (): LST at 8 days temporal and 1-km spatial resolution (MOD11A2) both for daytime and night-time conditions; NDVI at 16 days temporal and at 250-m spatial resolution (MYD13Q1).

The ESA (European Space Agency) Globcover land cover dataset (http://due.esrin.esa.int/globcover) was used based on 22 land cover classes as per the UN-FAO Land Cover Classification System at 300-m spatial resolution [21].

A nearest-neighbour resample procedure was applied using GDAL (Geospatial Data Abstraction Library) operators working on raster-tiles to homogenize the different spatial resolution of data layers. In particular, the monthly average LST and land-cover layers were initially resampled at 250-m resolution. Then the mean daytime and night-time LST was estimated pixel-wise by the corresponding mean NDVI value, also considering the land cover stratification, by applying two nested statistical model procedures: 1) the Linear Regression Model (LRM); 2) the Generalized Additive Model (GAM) [22]. LST model-based downscaling using NDVI as a predictor has already been used and discussed for specific environmental scenario purposes [9]. It is based on the existence of a significant negative correlation between LST and NDVI during the warm season [11]. In this study, the use of a nested procedure that involves only one single predictive model for each land-cover class, allows for avoiding the well-known biases of the large variability of surface emissivity in urban areas. Furthermore, the use of the GAM smoothing scheme allows for accounting for the non-linearity of the relationships between LST and NDVI during summer. Model performances and their respective accuracy were assessed through the following model metrics: mean coefficient of determination (R^2^); statistical model significance; root-mean square error (RMSE). In addition, the linear regression coefficient was also calculated. The RMSE represents a good measure of model accuracy and is widely used to assess the accuracy of spatial analysis and remote sensing data [23].

The same nearest-neighbour resample procedure was also used by averaging the monthly LST outcomes in summer (May-September) to obtain a high-resolution (100 m) product spatially consistent with the population layers (100 m). Water surfaces were excluded from the procedure. Geostatistical and GIS procedures were written in R-language using specific R-packages, such as rgdal, raster and mgcv, available online on: http://cran.r-project.org/ (packages link). Demonstrative work-flow, data and code functions built-up for this work are available on github.com/meteosalute/mapheatrisk.

### Exposure and vulnerability layers: total and elderly population

The exposure layer was represented by the population density data extracted from the JRC population grid (version 5 available on http://www.eea.europa.eu/data-and-maps/data/population-density-disaggregated-with-corine-land-cover-2000-2) which entails the 1-km^2^ population density for 2001 (source Eurostat) on a 100-m grid for the European Union. Population by commune was disaggregated with the CORINE land cover 2000 using the downscaling method described in Gallego [18]. This product was further enhanced by stratifying the total population grid by age classes, using the Local Administrative Units 2 (LAU2) data, formerly the Nomenclature of Territorial Units for Statistics (NUTS) level 5, which in Italy correspond to municipalities and are available on the Eurostat website (http://epp.eurostat.ec.europa.eu). We represented the vulnerability layer by the aggregated age class of people aged 65 or over. For each city, the vulnerable population layer (people > 65 years) was calculated by multiplying the cumulative percentage of the vulnerable population for the pixel-wise total population layer. In this way, the consistency of the elderly was obtained numerically and spatially in compliance with the LAU2 European statistical data (Eurostat) (S1 Table).

### Heat-related elderly risk mapping

**Fig 1:**
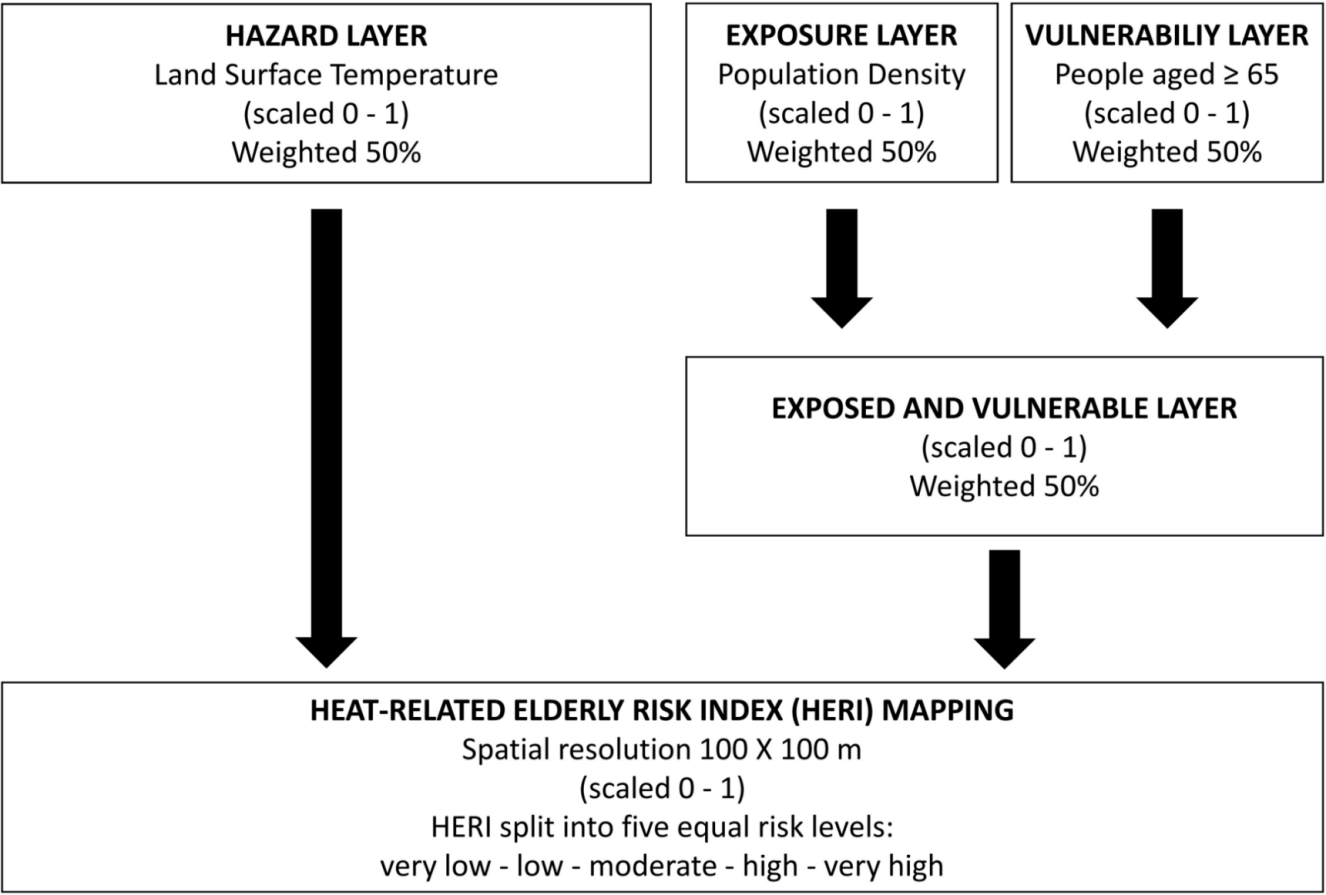
Work-flow of the spatial urban-hazard risk analysis employed to develop the final mapping of the Heat-related Elderly Risk Index (HERI).

The spatial methodology employed to provide heat-related health risk mapping envisaged a normalization procedure, followed by a weighted-layers combining procedure and development of the final mapping of the HERI (Fig 1). The normalization was used to obtain the hazard, exposure and vulnerability layers on the same scale (0 to 1) by dividing each value of an individual layer by the range of variability.

The following step was the combination of the normalized layers through a weighting procedure. To avoid subjective manipulation, all weightings were kept equal. In particular, the exposure and vulnerability layers were combined in a single “exposed and vulnerable” layer (each weighted at 50%) which was then spatially combined with the hazard layer (weighted at 50%). This spatial methodology follows the one successfully applied in previous recent studies [14, 15]. In short, HERI varying between 0 and 1 was obtained. The final city-specific mapping visualization was created by splitting the HERI into five equal-risk levels: very low (HERI ≤ 0.2), low (0.2 < HERI ≤ 0.4), moderate (0.4 < HERI ≤ 0.6), high (0.6 < HERI ≤ 0.8), and very high (HERI > 0.8).

The percentage of the urban coverage area for each HERI level was also assessed.

## Results

### Daytime and night-time LST estimation

The most representative land cover classes, showing average percentage surface areas greater than 10% of the total city area, were C20 (mean value of 24.1% among all cities), C130 (19.6%), C50 (18.1%), C14 (16.8%) and C190 (13.1%). The C190 land cover class is of great interest because it describes all the areas that have an artificial cover as a result of human activities such as construction (cities, towns, transport), extraction (open mines and quarries) or waste disposal. The C190 showed the highest percentage of coverage area values in cities with the greatest total population density (> 4,000 total population per km^2^) such as Milan, Naples, Turin and Palermo (S1 and S2 Tables).

The estimation of daytime and night-time LST through the LRM procedure always showed negative and generally significant (p<0.05) LST-NDVI regression coefficients for every land cover class (Tables 1 and 2). However, a few non-significant relationships (C90 and C110 during daytime in Table 1 and C11, C70 and C130) were also found.

**Table 1:** Summary of the metrics of Linear Regression and Generalized Additive Models during daytime per land cover classes.

GlobCover Land Cover classes	LST-NDVI^a^ Linear Regression Model				LST-NDVI^a^ Generalized Additive Model		
	R^2^	Regression coefficients	Significance	RMSE^b^	R^2^	Significance	RMSE^b^
C11	0.17	-5.23	0.0137	0.95	0.19	0.0089	0.93
C14	0.23	-11.82	0.0313	1.70	0.29	0.0000	1.61
C20	0.25	-12.46	0.0391	1.91	0.28	0.0113	1.84
C50	0.24	-16.00	0.0300	1.89	0.27	0.0000	1.84
C70	0.16	-11.52	0.0486	2.17	0.23	0.0151	2.04
C90	0.03	-6.45	0.2636	1.85	0.10	0.0728	1.68
C100	0.16	-13.15	0.0041	2.14	0.20	0.0010	2.04
C110	0.07	-1.07	0.0846	1.82	0.25	0.0011	1.44
C120	0.22	-27.62	0.0002	2.92	0.32	0.0000	2.54
C130	0.11	-7.28	0.0382	2.21	0.15	0.0147	2.13
C150	0.20	-10.17	0.0206	2.02	0.28	0.0009	1.88
C190	0.08	-3.50	0.0201	2.12	0.12	0.0075	2.07
Average	0.18	-10.28	0.0278	1.99	0.23	0.0068	1.89

^a^LST-NDVI: Land Surface Temperature-Normalized Difference Vegetative Index

^b^RMSE: Root-mean square error.

**Table 2:** Summary of the metrics of Linear Regression and Generalized Additive Models during night-time per land cover classes.

GlobCover Land Cover classes	LST-NDVI^a^ Linear Regression Model				LST-NDVI^a^ Generalized Additive Model		
	R^2^	Regression coefficients	Significance	RMSE^b^	R^2^	Significance	RMSE^b^
C11	0.09	-2.34	0.0682	0.57	0.14	0.0000	0.55
C14	0.10	-0.84	0.0273	0.71	0.15	0.0042	0.68
C20	0.08	-1.23	0.0316	0.77	0.12	0.0128	0.74
C50	0.09	-3.86	0.0408	0.83	0.12	0.0142	0.80
C70	0.10	-2.30	0.0614	0.93	0.15	0.0109	0.89
C90	0.04	-2.80	0.0026	0.84	0.10	0.0008	0.80
C100	0.07	-0.85	0.0425	1.15	0.12	0.0058	1.08
C110	0.17	-6.66	0.0000	1.13	0.25	0.0000	1.03
C120	0.20	-10.33	0.0000	1.22	0.30	0.0000	1.04
C130	0.06	-2.21	0.0639	1.03	0.08	0.0413	1.01
C150	0.08	-1.80	0.0303	0.81	0.12	0.0208	0.79
C190	0.12	-3.90	0.0047	1.04	0.13	0.0001	1.03
Average	0.09	-2.40	0.0346	0.88	0.13	0.0136	0.85

^a^LST-NDVI: Land Surface Temperature-Normalized Difference Vegetative Index.

^b^RMSE: Root-mean square error.

LST estimations assessed through the GAM procedure performed better than the LST assessed thorough the LRM approach (Tables 1 and 2). The GAM procedure always showed higher R^2^ values than those observed for LRM. The accuracy of the LST estimation was better when the GAM procedure was applied: the RMSE-related GAM procedure was always lower than the RMSE-related LRM approach in both daytime (Table 1) and night-time (Table 2) periods. Furthermore, a higher accuracy of the night-time LST estimation (average night-time RMSE = 0.88 and 0.85 for LRM and GAM respectively) than the daytime LST (average daytime RMSE = 1.99 and 1.89 for LRM and GAM respectively) (Tables 1 and 2) was always observed for each land cover class.

### High-resolution daytime heat-related elderly risk summer maps

**Fig 2:**
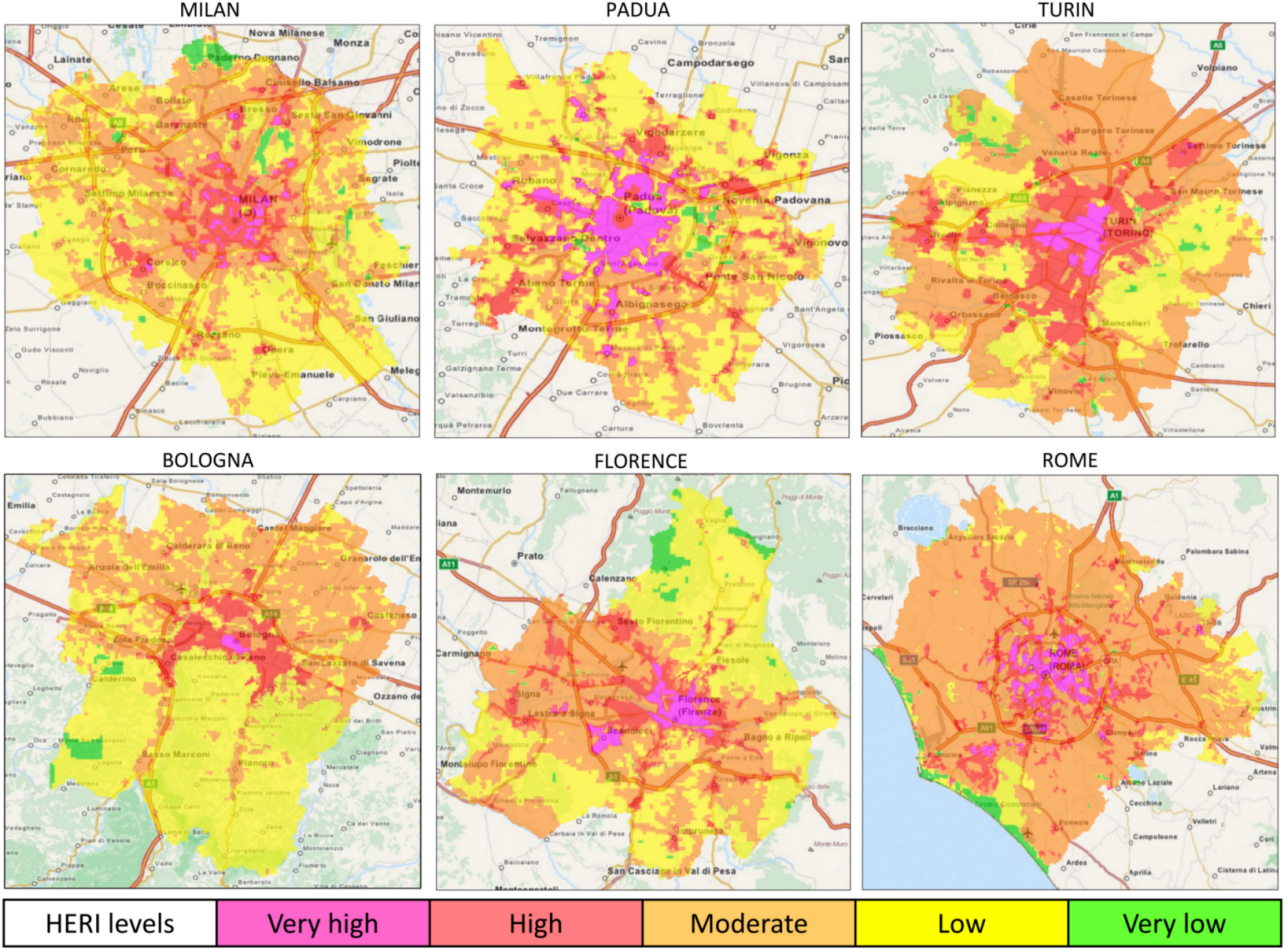
Maps of daytime heat-related elderly risk levels in the main inland Italian cities during the 2001–2013 summers (May-September). HERI: Heat-related Elderly Risk Index.

**Fig 3:**
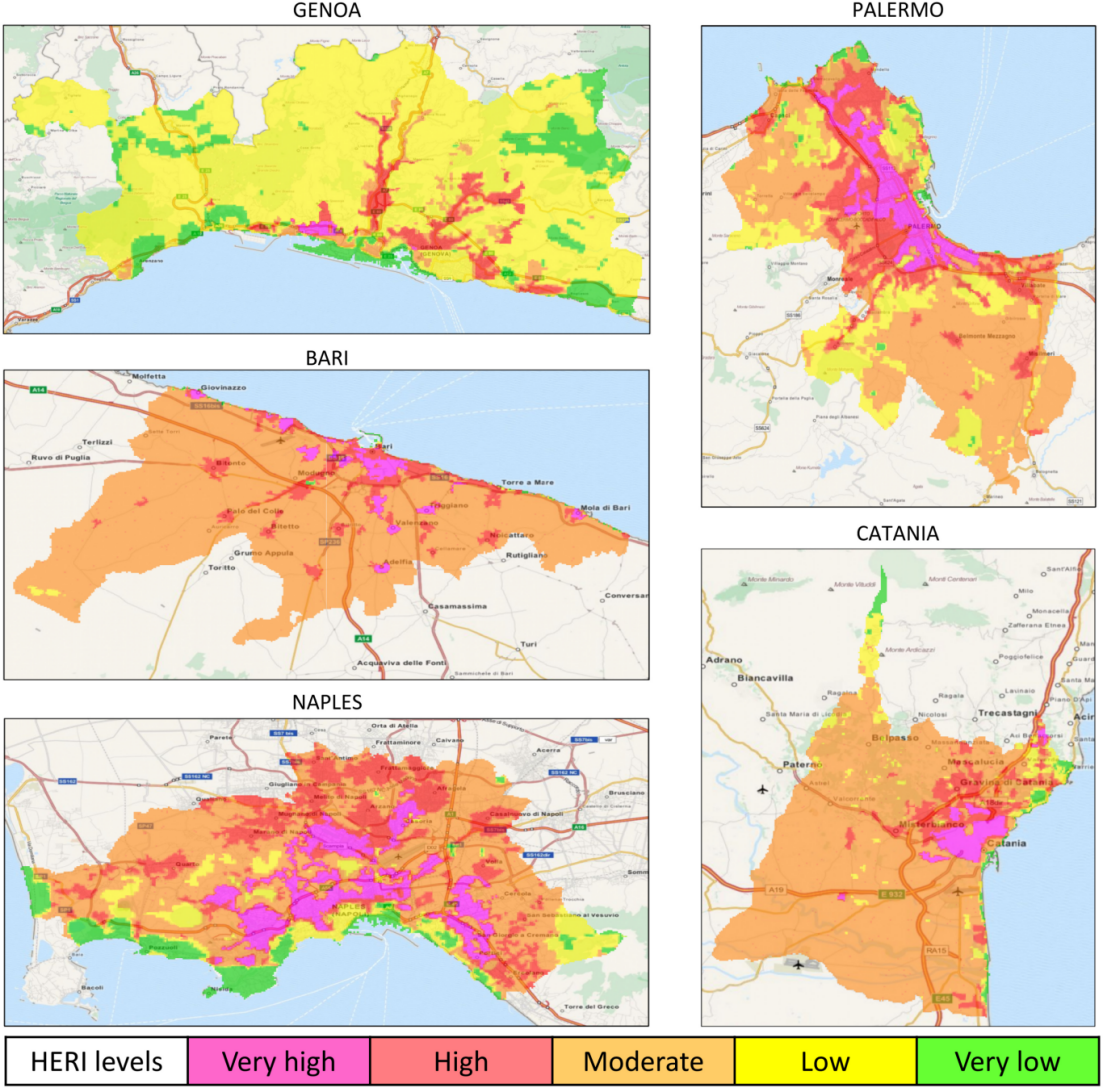
Maps of daytime heat-related elderly risk levels in the main coastal Italian cities during the 2001–2013 summers (May-September). HERI: Heat-related Elderly Risk Index.

The results demonstrated a heterogeneous intra-urban variation of daytime HERI levels in both inland (Fig 2) and coastal (Fig 3) cities.

The highest mean LST (Tables 3 and 4) was observed in the coastal city of Catania (32.2°C at the moderate HERI level), followed by Rome (31.3°C at the very high risk level), with the lowest LST values in the coastal cities of Genoa and Catania, with 11.0°C and 12.3°C respectively at the very low risk level. Most cities (3 inland and 3 coastal) showed the highest mean LST value at the moderate risk level, and several cities (3 inland and 1 coastal) at the very high or high (1 coastal city) risk levels (Tables 3 and 4).

**Table 3:** Daytime heat-related elderly risk index levels for the most populous inland Italian cities during the 2001–2013 summers (May-September).

Cities	HERI^a^ levels	Mean LST^b^ (°C) ±SD^c^	Coverage area (%)	Population frequency (%)		Population density (Pop. per km^2^)	
				Total	≥ 65	Total	≥ 65
Turin	Very Low	12.5(±0.5)	0.4	0.0	0.0	4.6	0.8
	Low	18.4(±2.0)	27.4	1.2	1.1	91.0	13.7
	Moderate	22.6(±1.2)	55.0	15.8	14.5	622.2	94.3
	High	22.2(±1.6)	14.6	60.3	59.9	8,915.3	1,464.4
	Very high	20.8(±2.0)	2.6	22.7	24.5	18,598.5	3,320.6
Milan	Very Low	20.4(±0.2)	1.8	0.7	0.6	1,580.9	231.1
	Low	23.8(±0.6)	49.1	2.4	2.4	199.8	32.3
	Moderate	24.3(±0.4)	39.3	58.8	56.8	6,140.0	947.8
	High	24.6(±0.2)	8.4	31.3	32.8	15,249.2	2,564.2
	Very high	24.8(±0.1)	1.4	6.8	7.4	19,313.8	3,333.8
Padua	Very Low	20.8(±1.6)	0.5	0.1	0.1	110.2	18.3
	Low	25.7(±0.5)	48.7	2.8	2.5	61.9	8.0
	Moderate	26.5(±0.6)	33.2	8.0	7.1	251.4	33.2
	High	26.1(±0.8)	8.2	34.4	30.8	4,376.9	581.4
	Very high	26.3(±0.3)	9.4	54.7	59.5	6,102.8	984.8
Bologna	Very Low	20.2(±0.8)	1.2	0.1	0.1	50.2	9.4
	Low	25.7(±1.7)	55.0	4.2	3.8	66.6	12.4
	Moderate	28.6(±0.7)	37.0	24.1	22.6	564.2	106.9
	High	28.1(±0.7)	6.5	66.4	68.1	8,907.5	1,851.0
	Very high	27.9(±0.4)	0.3	5.2	5.4	15,634.3	3,303.9
Florence	Very Low	20.2(±1.3)	2.1	0.0	0.0	19.5	3.3
	Low	24.7(±1.2)	44.3	2.1	2.0	52.3	9.8
	Moderate	27.5(±0.7)	41.7	4.4	4.4	118.4	22.7
	High	28.2(±0.6)	8.7	56.4	55.6	7,231.0	1,368.4
	Very high	28.6(±0.3)	3.2	37.1	38.0	12,886.6	2,535.5
Rome	Very Low	13.2(±2.4)	0.2	0.0	0.0	30.4	3.6
	Low	24.9(±1.5)	8.2	0.4	0.3	61.6	7.9
	Moderate	30.4(±1.2)	76.9	6.6	6	109.3	14.7
	High	31.2(±0.7)	10.8	58.6	58.3	6,889.3	1,021.2
	Very high	31.3(±0.3)	3.9	34.4	35.4	11,237.0	1,723.5

^**a**^HERI: Heat-related Elderly Risk Index.

^b^LST: Land Surface Temperature.

^c^SD: Standard Deviation.

**Table 4:** Daytime heat-related elderly risk levels for the most populous coastal Italian cities during the 2001–2013 summers (May-September).

Cities	HERI^a^ levels	Mean LST^b^ (°C) ±SD^c^	Coverage area (%)	Population frequency (%)		Population density (Pop. per km^2^)	
				Total	≥ 65	Total	≥ 65
Genoa	Very Low	11.0(±1.5)	8.7	0.1	0.1	15.5	3.4
	Low	15.5(±1.9)	81.4	5.3	5.4	81.8	17.4
	Moderate	19.3(±2.5)	3.6	9.2	9.6	3,344.5	719.3
	High	20.0(±1.8)	5.8	74.0	73.6	15,976.2	3,296.8
	Very high	18.4(±0.9)	0.5	11.4	11.3	27,449.1	5,660.1
Naples	Very Low	12.8(±2.6)	0.7	0.0	0.0	67.6	7.9
	Low	22.7(±1.9)	8.0	0.2	0.2	108.3	11.5
	Moderate	27.9(±1.5)	56.0	8.5	7.1	867.3	77.5
	High	28.6(±1.8)	20.0	39.3	31.9	11,253.2	960.8
	Very high	28.9(±0.9)	15.3	52.0	60.8	19,496.8	2,399.2
Bari	Very Low	16.3(±3.1)	0.0	0.0	0.0	0	0
	Low	25.0(±1.1)	0.3	0.0	0.0	38.2	4.1
	Moderate	30.4(±0.4)	88.9	9.2	8.5	86.6	10.4
	High	30.1(±0.7)	7.6	56.1	54.5	6,175.5	778.0
	Very high	30.2(±0.4)	3.2	34.7	37.0	9,075.2	1,254.5
Palermo	Very Low	16.6(±2.2)	0.2	0.0	0.0	191.5	25.4
	Low	25.6(±1.1)	20.8	0.6	0.6	64.8	7.9
	Moderate	28.1(±1.0)	52.9	2.6	2.7	114.2	13.8
	High	27.8(±0.7)	17.8	52.3	52.1	6,824.6	809.5
	Very high	28.0(±0.4)	8.3	44.5	44.6	12,403.0	1,478.9
Catania	Very Low	12.3(±3.9)	0.4	0.0	0.0	7.9	2.8
	Low	26.2(±3.3)	4.7	0.4	0.5	92.8	14.1
	Moderate	32.2(±1.4)	84.1	19.1	16.3	257.7	28.2
	High	31.5(±2.2)	7.2	42.8	41.1	6,705.9	822.2
	Very high	32.0(±0.6)	3.6	37.7	42.1	11,722.8	1,672.8

^**a**^HERI: Heat-related Elderly Risk Index.

^b^LST: Land Surface Temperature.

^c^SD: Standard Deviation.

The moderate risk level also covered most of the total surface urban area (>50%) in Rome and Turin (Table 3) and almost all coastal cities (with one exception, Table 4). The remaining four inland cities and the coastal city of Genoa showed the low risk level with the highest coverage area (Tables 3 and 4). The hazardous HERI level (very high risk) was generally localized to encompass the city-centre in inland cities (Fig 2), and the inner area in coastal cities (Fig 3), except for Genoa (Fig 3). The very high risk had the largest coverage area in Naples (about 15%, Table 4), followed by Padua (9.4%) and the coastal city of Palermo (8.3%). The lowest coverage areas were observed in Bologna (0.3%; Table 3) and Genoa (0.5%; Table 4).

The two most dangerous HERI levels were characterized in all cities by the highest total and elderly population frequency and density. The population density generally showed a progressive increase from the very low to the very high risk level (Tables 3 and 4). The population frequency generally showed the maximum at the high risk level (Tables 3 and 4) and in one case (Milan in Table 3) at the moderate HERI level.

### High-resolution night-time heat-related elderly risk summer maps

**Fig 4:**
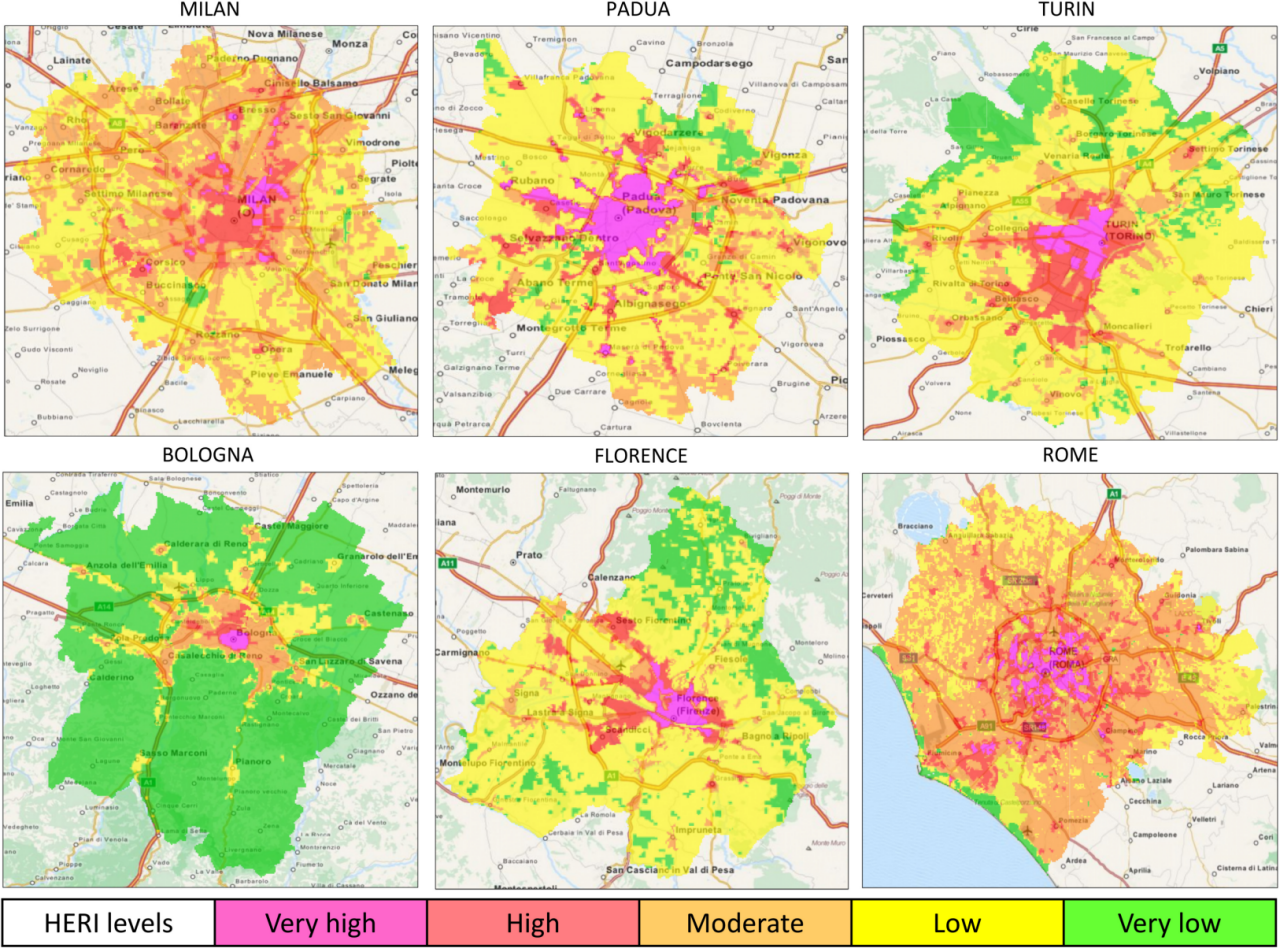
Maps of night-time heat-related elderly risk levels in the main inland Italian cities during the 2001–2013 summers (May-September). HERI: Heat-related Elderly Risk Index.

**Fig 5:**
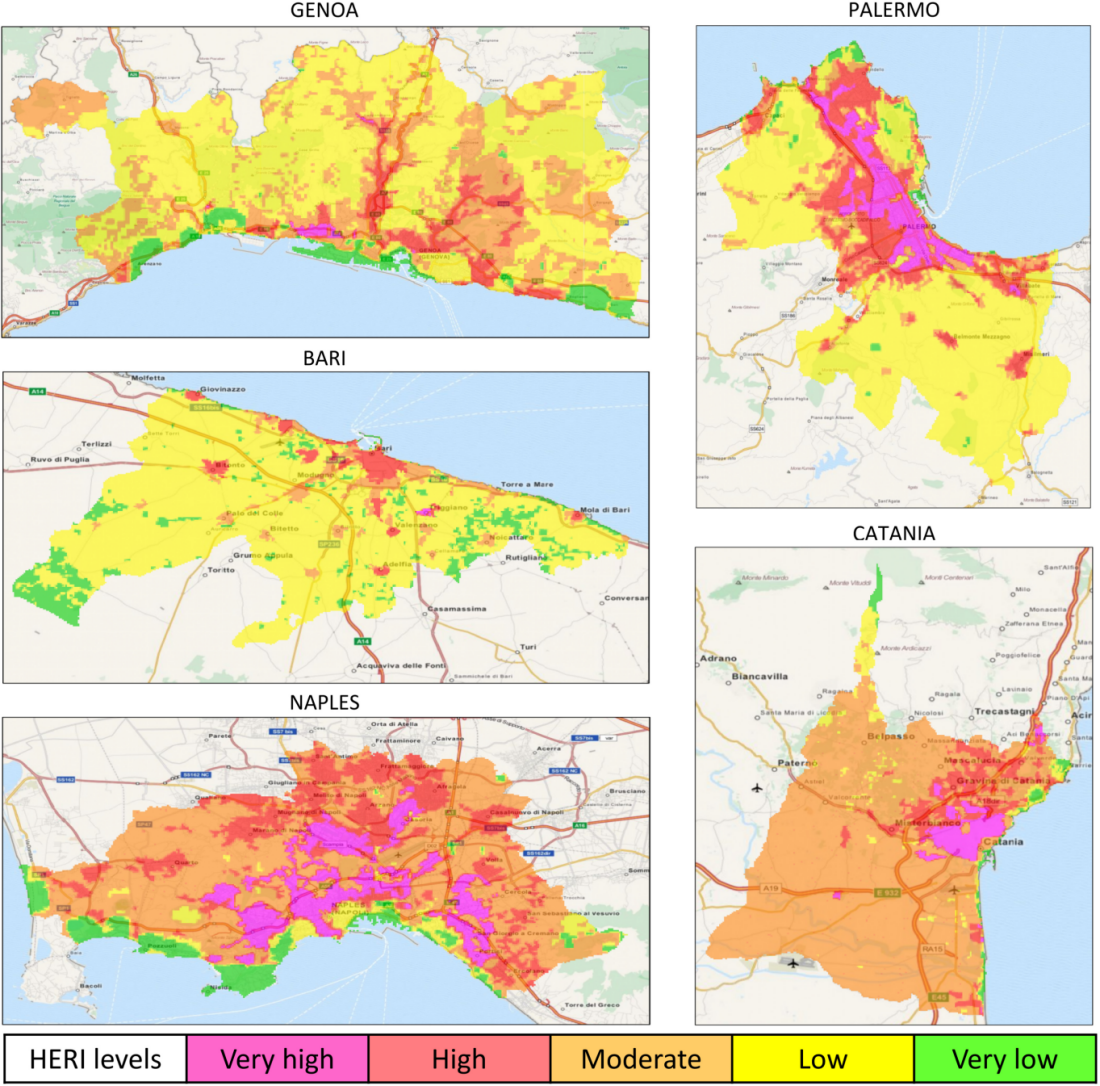
Maps of night-time heat-related elderly risk levels in the main coastal Italian cities during the 2001–2013 summers (May-September). HERI: Heat-related Elderly Risk Index.

The results demonstrated a heterogeneous intra-urban variation of night-time HERI levels in both inland (Fig 4) and coastal (Fig 5) cities.

Most of cities showed a progressive rise in the mean LST from the lowest value at the very low level to the highest value at the very high risk level. In particular, the highest mean LST was observed in Bologna (18.6°C), followed by Florence (17.6°C) and Rome (17.5°C) (Table 5). Among coastal cities, Palermo showed the highest mean LST (17.1°C) (Table 6) while Genoa (1.9°C) and Catania (3.2°C) had the lowest LST values.

**Table 5:** Night-time heat-related elderly risk levels for the most populous inland Italian cities during the 2001–2013 summers (May-September).

Cities	HERI^a^ levels	Mean LST^b^ (°C) ±SD^c^	Coverage area (%)	Population frequency (%)		Population density (Pop. per km^2^)	
				Total	≥ 65	Total	≥ 65
Turin	Very Low	6.4(±0.5)	15.5	0.3	0.3	45.6	6.3
	Low	8.4(±0.8)	62.2	9.5	8.1	327.6	46.3
	Moderate	9.5(±1.1)	11.9	34.1	33.0	6,229.5	996.2
	High	10.8(±0.7)	7.8	34.0	34.8	9,455.0	1,602.3
	Very high	11.4(±0.8)	2.6	22.1	23.8	18,700.2	3,327.7
Milan	Very Low	8.1(±0.2)	0.5	0.0	0.0	47	6.4
	Low	10.5(±0.6)	45.8	2.4	2.0	216.7	29.0
	Moderate	11.0(±0.5)	44.7	60.6	58.7	5,550.2	859.2
	High	11.0(±0.6)	7.7	30.7	32.5	16,256.0	2,761.7
	Very high	11.7(±0.1)	1.3	6.3	6.8	19,333.2	3,336.6
Padua	Very Low	8.8(±0.2)	5.5	0.3	0.3	55.7	7.1
	Low	10.3(±0.5)	63.6	4.2	3.6	68.4	8.8
	Moderate	11.0(±0.5)	15.0	12.4	10.8	860.0	111.4
	High	10.7(±0.5)	7.5	34.4	30.9	4,807.9	639.9
	Very high	11.2(±0.3)	8.4	48.7	54.4	6,061.9	1,003.4
Bologna	Very Low	15.7(±0.3)	83.1	7.9	6.9	82.5	14.5
	Low	17.1(±0.6)	9.5	16.5	16	1,506.1	294.9
	Moderate	17.3(±0.5)	6.0	57.5	58.5	8,241.7	1,703.8
	High	18.1(±0.4)	0.9	9.6	9.9	9,256.0	1,918.4
	Very high	18.6(±0.5)	0.5	8.5	8.7	15,633.9	3,240.1
Florence	Very Low	14.5(±0.2)	17.3	0.5	0.4	30.6	5.5
	Low	15.5(±0.6)	68.2	5.0	4.9	82.0	15.3
	Moderate	16.6(±0.9)	6.4	23.8	23.7	4,096.0	783.0
	High	17.1(±0.4)	5.7	41.5	40.8	8,075.4	1,522.5
	Very high	17.6(±0.4)	2.4	29.2	30.2	13,704.5	2,725.7
Rome	Very Low	7.1(±1.3)	0.2	0.0	0.0	24.2	2.9
	Low	15.0(±0.5)	26.0	1.1	1.0	55.7	7.5
	Moderate	16.0(±0.6)	59.4	6.8	6.1	145.7	19.4
	High	16.8(±0.6)	10.6	58.2	57.9	6,983.6	1,037.0
	Very high	17.5(±0.6)	3.8	33.9	35.0	11,256.6	1,728.5

^**a**^HERI: Heat-related Elderly Risk Index.

^b^LST: Land Surface Temperature.

^c^SD: Standard Deviation.

**Table 6:** Night-time heat-related elderly risk levels for the most populous coastal Italian cities during the 2001–2013 summers (May-September).

Cities	HERI^a^ levels	Mean LST^b^ (°C) ±SD^c^	Coverage area (%)	Population frequency (%)		Population density (Pop. per km^2^)	
				Total	≥ 65	Total	≥ 65
Genoa	Very Low	1.9(±1.9)	0.1	0.0	0.0	58.6	13.8
	Low	10.6(±0.6)	60.2	1.6	1.6	34.1	7.0
	Moderate	12.0(±0.6)	32.7	8.4	8.7	323.6	69.4
	High	12.4(±1.2)	6.4	77.4	77.1	15,269.7	3,156.8
	Very high	11.7(±1.0)	0.6	12.6	12.6	26,094.7	5,416.5
Naples	Very Low	7.0(±1.6)	0.3	0.0	0.0	550.0	55.7
	Low	12.2(±1.3)	2.8	0.1	0.1	142.4	18.0
	Moderate	14.9(±0.7)	60.3	7.1	6.0	676.7	60.1
	High	15.4(±0.9)	20.9	39.8	32.1	10,910.5	927.0
	Very high	15.6(±0.5)	15.7	53.0	61.8	19,397.8	2,383.3
Bari	Very Low	12.7(±0.4)	10.8	1.1	1	85.3	10.0
	Low	13.8(±0.4)	79.1	11.4	10.4	120.3	14.3
	Moderate	14.6(±0.6)	6.2	45.4	44.7	6,077.8	775.7
	High	15.1(±0.5)	3.8	40.5	42.5	8,844.9	1,201.2
	Very high	15.2(±0.3)	0.1	1.6	1.4	13,200.0	1,547.3
Palermo	Very Low	11.5(±0.6)	0.1	0.0	0.0	0	0
	Low	14.4(±0.5)	65.2	1.2	1.3	43.2	5.5
	Moderate	16.0(±1.0)	10.5	7.5	8.1	1,654.7	212.5
	High	16.5(±0.7)	15.8	46.3	45.9	6,815.6	803.2
	Very high	17.1(±0.6)	8.4	45.0	44.7	12,341.2	1,460.6
Catania	Very Low	3.2(±1.1)	0.2	0.0	0.0	0	0
	Low	10.6(±1.8)	4.0	0.1	0.1	18.8	3.2
	Moderate	15.0(±0.8)	83.5	13.6	11.6	184.5	20.2
	High	15.2(±0.7)	8.0	43.3	40.2	6,130.4	726.4
	Very high	15.6(±0.8)	4.3	43.0	48.1	11,221.4	1,604.6

^**a**^HERI: Heat-related Elderly Risk Index.

^b^LST: Land Surface Temperature.

^c^SD: Standard Deviation.

The low risk level had the highest coverage area in most of cities (7 cities; Tables 5 and 6), while Bologna showed the highest absolute coverage area value (83.1%) coinciding with the very low risk level. However, the moderate risk also revealed the highest coverage area in Rome (Table 5) and two coastal cities (Naples and Catania) (Table 6). The hazardous risk level was generally localized to encompass the city-centre in inland cities (Fig 4), except for Milan, mostly localized in the Northeast urban area. The very high risk level was localized in the inner coast of coastal cities, except for Genoa and Bari (Fig 5). Naples had the greatest coverage area for the very high risk level (15.7%; Table 6), followed by another coastal city (Palermo) and the inland city of Padua (8.4%; Table 5). The other cities showed the worst risk level ranging from 1% (Milan) to about 4% (Rome and Catania). The lowest coverage areas (<1%) were observed in two coastal cities, Genoa and Bari (Table 6), and the inland city of Bologna (Table 5).

The total and elderly population density showed a progressive rise from very low to very high risk levels (Tables 5 and 6). However, most of cities showed the population frequency that reached the maximum when the moderate or the high risk levels occurred (Tables 5 and 6).

## Discussion

This multi-city study provides a detailed intra-city description of heat-related elderly risk variability. It is a valid contribution to previous epidemiological heat-related health studies.

The main contribution of this work is the identification of high-resolution urban areas characterized by potentially different heat-risk levels for the health of vulnerable people, i.e. the elderly. Critical heat-risk urban areas are identified with up-to-street-level details (S2 Fig). This information might potentially enable effective short-term (i.e. allocation of water supply, temporary health services, preparation of specific transport to cooling centres) and medium/long-term (i.e. encouraging greenings area, modification of buildings surfaces) intervention strategies by local authorities, urban planners and health agencies when deciding heat-related health prevention actions at the small-urban-area level.

The innovation of this study is its ability to use a 13-year spatio-temporal series of remote sensing MODIS LST data downscaled at high-resolution (100 m). This variable is commonly adopted to characterize the UHI effect [24], recently identified as an emerging trend in public health [25].

This study confirms the negative LST-NDVI relationship of land cover classes highlighted in previous studies, especially during the summer months [9, 10, 11]. Furthermore, the introduction of the GAM procedure has allowed for considering the potential non-linearity of the LST-NDVI relationship, as indicated by other authors [11]. This study also provides an additional contribution by revealing a higher accuracy (RMSE reduction) of LST estimation via use of the GAM procedure instead of the LST assessed through the LRM approach.

For the first time LST was combined with high-resolution (100-m grid) exposure and vulnerability layers assessed by using population density grids (total and elderly population) spatially available over a wide geographical area [18].

The decision to focus on urban areas stems from recent extreme-heat events causing serious health, economic and social problems worldwide (Europe, USA, Australia and Asia) with strong heat-impact due to global warming predicted in cities [26]. In addition, the World Urbanization Prospects [27] estimates that about 70% of Italy’s total population residing in urban areas is expected to rise to 80% by 2050. Furthermore, according to recent estimates, about 33% of Italy’s population will be over 65 by 2050 against approximately 21% reported in the 2014 census (http://demo.istat.it/pop2014/index.html). Therefore, an increasing number of more vulnerable individuals is expected to become exposed to future heat events and detailed methodologies are indispensable for assessing the intra-city heat-related elderly risks.

The highest heat-related vulnerability of the elderly is due to physiological, health and socio-economical status [28]. Consequently, many European and all major Italian cities have developed specific heat health warning systems (HHWSs) for the elderly. Recent studies have also revealed a reduction in heat-related elderly mortality in Italian cities after the establishing HHWSs [29, 30]. Future implementation of intra-city HERI in existing HHWSs might further help reduce the heat-effect impact on the health of most the vulnerable. However, HERI maps should also be updated each year and include the latest available remote sensing MODIS data and population density grid based on the most recent population census. In addition, the HERI could also be provided on a different temporal scale, i.e. monthly.

In this study, all areas characterized by the hazardous risk level also have the highest total and elderly population densities. For this reason, healthcare should immediately be provided in these zones in case of excessive heat events. Most cities showed the hazardous HERI level in inner-city areas, which is in agreement with previous studies showing that the heat effect was mainly evident among people living in inner-city neighbourhoods [14, 31], also caused by exposure to the combined effects of UHI and air pollution.

Interestingly, urban areas with the hazardous HERI level were not necessarily characterized by the highest daytime LST. Conversely, there was a night-time correspondence between the harmful HERI level and the highest LST, in agreement with the fact that UHI is a marked night-time phenomenon [6] when energy accumulated during the day is released into the atmosphere. On the other hand, a larger temperature daytime variability is observed due to the variable sunlight contribution, which increases the heterogeneity when LST is measured in shadow or direct sunlight [32]. This is also supported by the higher accuracy (the lowest RMSE value) of the night-time LST estimation than the daytime LST observed for every land cover class investigated in the study.

The urban areas affected by the two most dangerous HERI levels were greater in coastal (the average from diurnal and nocturnal periods was 11.3% and 6.0% for the high and very high risk levels respectively) than inland (8.1% and 3.3% for the high and very high risk levels respectively) cities. This geographical heterogeneity is particularly interesting because it is well known that tolerance to temperature extremes varies regionally [33] according to the population and its preparedness for heat conditions, and the local temperatures and frequency of extreme events. However, the ways in which city dwellers adapt physiologically and technologically to heat have not been investigated in this urban hazard risk analysis. In a previous study [34], we also hypothesized that populations living in coastal plain cities with milder climate conditions and low daily temperature variations where extremely high temperatures are uncommon or occur infrequently, appeared more susceptible and less adaptable to sudden temperature changes and extremes. Furthermore, findings from this study also showed that several urban coastal populations might be more susceptible to potential heat effects due to the existence of extensive heat-related high and very high risk areas.

### Strengths and Limitations

This study represents a starting point for identifying areas requiring adaptation-planning strategies [35] such as alternative land-surface changes in cities, allocation of economical and technological resources to counteract heat, and health-personnel organisation to support the growing number of vulnerable people living in urban high-risk areas in cases of excessive heat events. These adaptation actions and the need for detailed intra-city heat-related elderly risk assessments are of primary importance for minimising adverse health effects associated with the significant and progressive temperature rises already observed in many cities due to climate change and urbanization.

Other strengths of this study are linked to the fact that well-known, referenced and free public remote sensing (satellite MODIS data) and demographic (Eurostat source) datasets were used for the HERI assessment. Furthermore, all computerized procedures were coded, and made freely available on github.com/meteosalute/mapheatrisk, by using a well-referenced open source software (R statistical software). These strengths enable easily replicable and comparable studies. In this regard, one of the main strengths is the potential geographical reproducibility of the study. All grid-raster data used are spatially continuous, thus allowing for future investigations in marginal and developing urban areas. Since the same demographic grid-data and satellite data are also available in all European Union countries, the heat-risk analysis assessed nationally in this study could easily be extended to the rest of Europe in future studies.

This study also has some limitations. Firstly, the LST was used as a hazard-layer measure for potential population exposure. However, the relationship between LST and ambient temperature is very complex [36] and strongly influenced by atmospheric conditions and surface properties. Furthermore, microscale site characteristics have a greater influence on LST than on air temperature [6]. Nevertheless, a recent validation study of satellite-derived LST with in situ ambient temperature measurements [25] concluded that LST can be considered a useful variable to better understand the spatial variation in heat exposure over long time frames. Because the existing urban meteorological networks generally only cover limited areas, remotely-sensed data represent a useful dataset for spatial heat vulnerability analysis and a proxy for heat exposure by city inhabitants [37].

Another limitation is the fact that the exposed and vulnerable layer was only represented by the total and elderly population density. Conversely, no other socio-demographic and health variables (i.e. physiological adaptation, pre-existing health conditions, air conditioning use) were considered as often spatially fractioned and when available, they only apply to very few cities. Furthermore, specific grid platforms for other high-resolution socio-demographic or health variables are still not available. However, as these variables become available as gridded datasets in the future, they could easily be implemented in the risk assessment methodology and provide an improved version of urban hazard maps.

## Conclusions

This study shows the great potential of combining geospatial technologies and spatial demographic characteristics within a simple and flexible conceptual framework in order to provide high-resolution urban mapping of daytime and night-time heat-related elderly risk index. This result was achieved thanks to an effective downscaling smoothing procedure (GAM approach) of thermal surface satellite MODIS data. The GAM procedure allowed for improved daytime and night-time LST estimations compared to the linear regression model approach normally used in previous studies.

HERI maps in major Italian cities generally showed the hazardous HERI level localized to encompass the city-centre in inland cities and the inner areas of coastal cities. Furthermore, the two most dangerous HERI levels were greater in the coastal than the inland cities. This information is a potentially useful tool for delineating daytime and night-time high-risk intra-urban areas with high—not necessarily the highest—temperatures, as well as the greatest total and elderly population density. In this way, potential areas for intervention are immediately identified, thus supporting public health operators and facilitating coordination for heat-related emergencies.

## Supporting Information

S1 Fig Geographical location of the main Italian cities studied (OpenstreetMap Contributors ODbL, tiles MapQuest Mapnik). (TIF)

S2 Fig Map of the high-resolution (100 m) urban Heat-related Elderly Risk Index (HERI) for Rome during the 2001–2013 summers (May-September) (OpenstreetMap Contributors ODbL, tiles MapQuest Mapnik). (TIF)

S1 Table Geographical and demographic characteristics of the main Italian cities studied. (DOCX)

S2 Table Percent of surface area (% of the total city area) covered by specific land cover classes of the main Italian cities studied. (DOCX)
