# Supplementary figures and images for: Urban-Hazard Risk Analysis: Mapping of Heat-Related Risks in the Elderly in Major Italian Cities
Source: PLoS One. 2015 May 18;10(5):e0127277. doi: 10.1371/journal.pone.0127277 (PMC4436225; doi:10.1371/journal.pone.0127277)

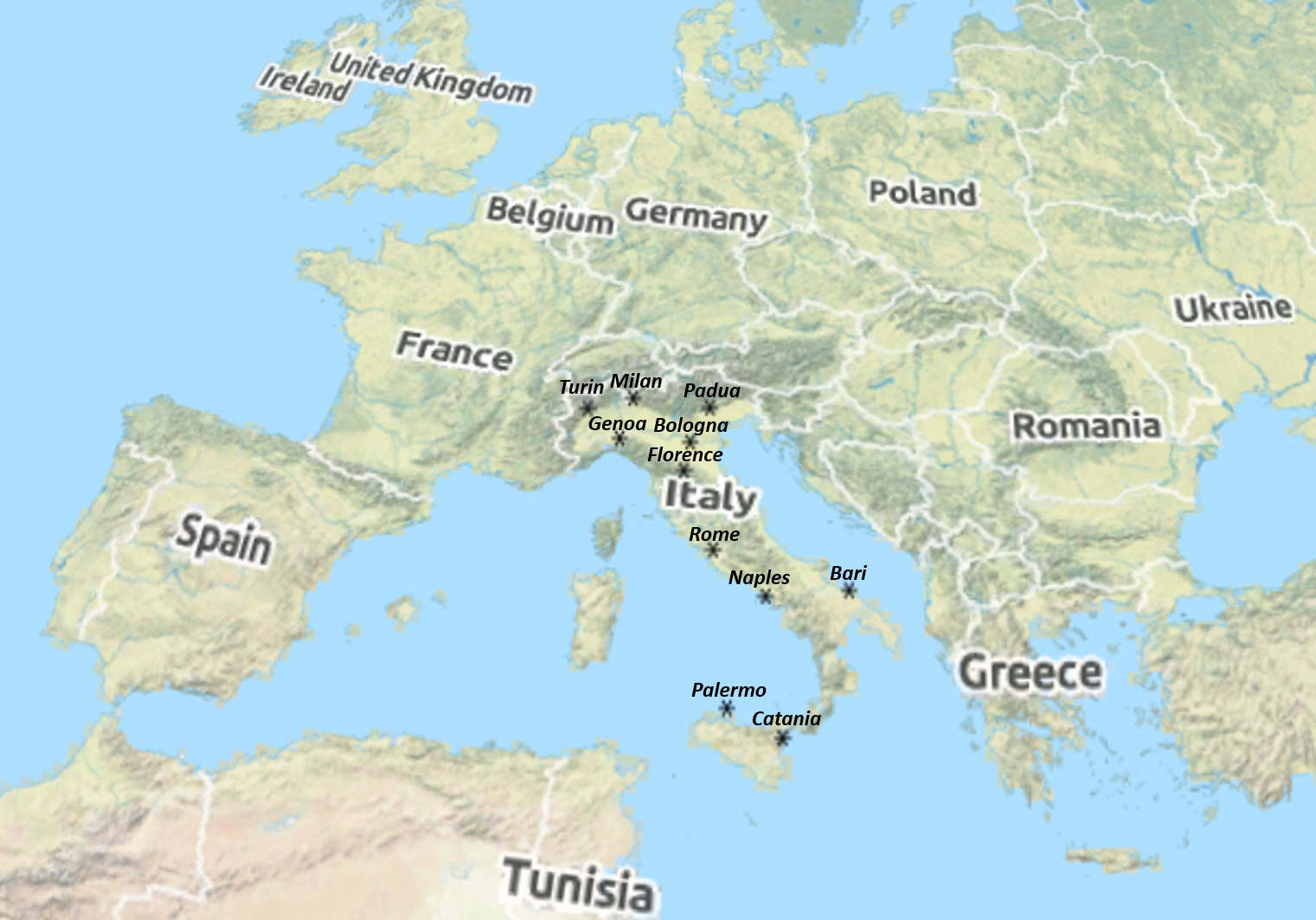

Supplement: S1 Fig — (TIF) [file pone.0127277.s001.tif]

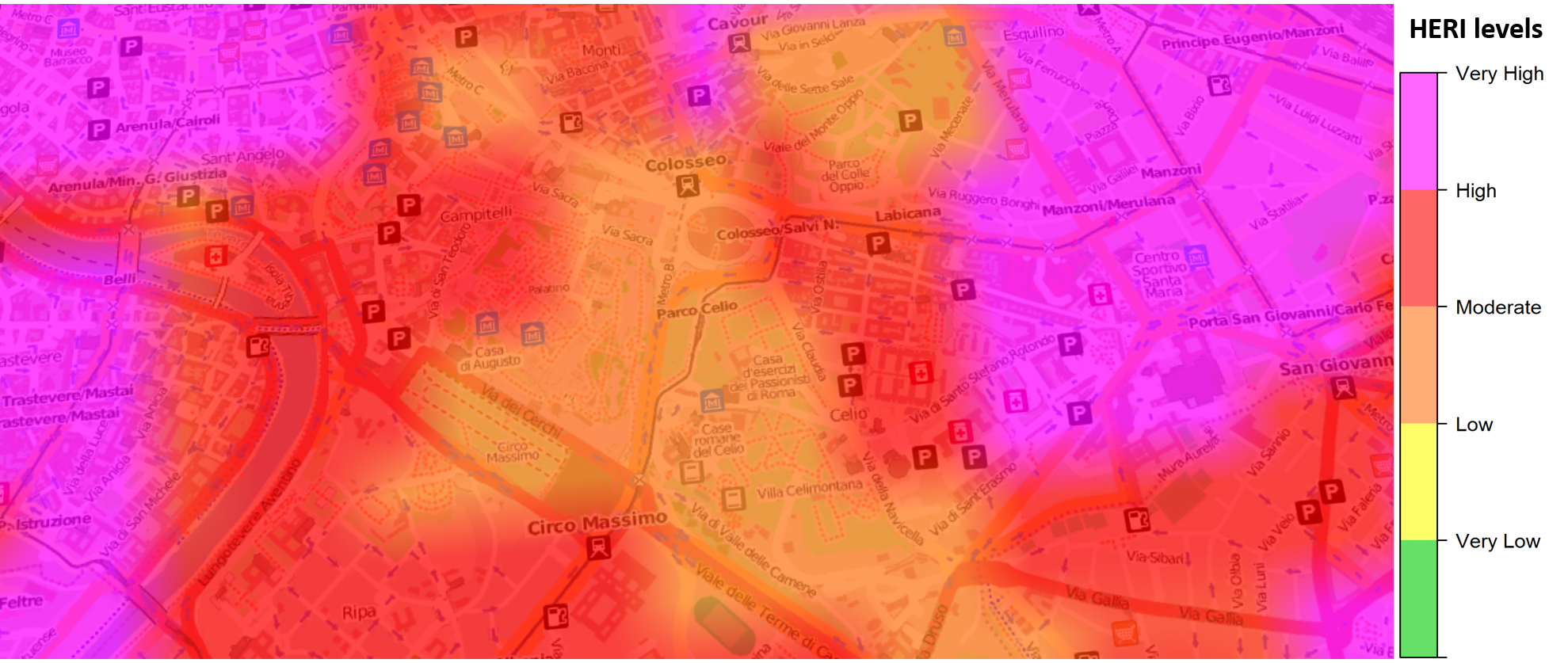

Supplement: S2 Fig — (TIF) [file pone.0127277.s002.tif]
