# Supplementary material for: Urban-Hazard Risk Analysis: Mapping of Heat-Related Risks in the Elderly in Major Italian Cities
Source: PLoS One. 2015 May 18;10(5):e0127277. doi: 10.1371/journal.pone.0127277 (PMC4436225; doi:10.1371/journal.pone.0127277)
Supplement: S1 Table — (DOCX) [file pone.0127277.s003.docx]

Table S1: Geographical and demographic characteristics of the main Italian cities studied.

| Cities and geographical coordinates | | Location | Surface area (km^2^) | Population count and  elderly frequency | | Population density  (Pop. per km^2^) | |
| --- | --- | --- | --- | --- | --- | --- | --- |
|  |  |  |  | Total | ≥ 65 | Total | ≥ 65 |
| Milan | 45°27′51″N  9°11′25″E  122 m a.s.l. | City-centre | 181.8 | 1,255,321 | 224,636 (17.9%) | 6,904.6 | 1,235.6 |
|  |  | Suburbs | 320.9 | 804,049 | 104,953 (13.1%) | 2,505.4 | 327.0 |
| Padua | 45°24′23″N 11°52′40″E  12 m a.s.l. | City-centre | 93.0 | 202,674 | 34,635 (17.1%) | 2,180.0 | 372.5 |
|  |  | Suburbs | 286.3 | 193,385 | 24,093 (12.5%) | 675.4 | 84.1 |
| Turin | 45°04′00″N  7°42′00″E  239 m a.s.l. | City-centre | 130.7 | 863,210 | 155,935 (18.1%) | 6,606.0 | 1,193.3 |
|  |  | Suburbs | 516.3 | 536,904 | 75,538 (14.1%) | 1,039.9 | 146.3 |
| Bologna | 44°29′38″N  11°20′34″E  54 m a.s.l. | City-centre | 141.2 | 370,976 | 79,013 (21.3%) | 2,626.6 | 559.4 |
|  |  | Suburbs | 482.0 | 168,203 | 30,213 (18.0%) | 349.0 | 62.7 |
| Genoa | 44°24′40″N  8°55′58″E  19 m a.s.l. | City-centre | 239.1 | 642,736 | 135,715 (21.1%) | 2,688.6 | 567.7 |
|  |  | Suburbs | 321.2 | 63,021 | 10,723 (17.0%) | 196.2 | 33.4 |
| Florence | 43°46′17″N  11°15′15″E  50 m a.s.l. | City-centre | 102.3 | 356,155 | 72,257 (20.3%) | 3,482.8 | 706.6 |
|  |  | Suburbs | 420.8 | 225,248 | 39,406 (17.5%) | 535.3 | 93.6 |
| Rome | 41°53′35″N  12°28′58″E  21 m a.s.l. | City-centre | 1,274.2 | 2,419,702 | 374,387 (15.5%) | 1,899.0 | 293.8 |
|  |  | Suburbs | 1,070.7 | 563,591 | 70,166 (12.4%) | 526.4 | 65.5 |
| Bari | 41°07′31″N 16°52′00″E  5 m a.s.l. | City-centre | 116.0 | 319,161 | 46,260 (14.5%) | 2,750.4 | 398.7 |
|  |  | Suburbs | 614.8 | 292,294 | 33,042 (11.3%) | 475.4 | 53.7 |
| Naples | 40°50′00″N  14°15′00″E  17 m a.s.l. | City-centre | 118.1 | 1,009,841 | 125,608 (12.4%) | 8,552.2 | 1,063.8 |
|  |  | Suburbs | 209.8 | 868,542 | 72,298 (8.3%) | 4,140.4 | 344.7 |
| Palermo | 38°06′56″N  13°21′41″E  14 m a.s.l. | City-centre | 160.1 | 688,649 | 81,809 (11.9%) | 4,300.8 | 510.9 |
|  |  | Suburbs | 176.2 | 91,040 | 10,958 (12.0%) | 516.7 | 62.2 |
| Catania | 37°30′10″N  15°05′14″E  7 m a.s.l. | City-centre | 181.8 | 323,642 | 46,587 (14.4%) | 1,780.4 | 256.3 |
|  |  | Suburbs | 321.1 | 246,696 | 26,311 (10.7%) | 768.3 | 81.9 |
