# Supplementary material for: Urban-Hazard Risk Analysis: Mapping of Heat-Related Risks in the Elderly in Major Italian Cities
Source: PLoS One. 2015 May 18;10(5):e0127277. doi: 10.1371/journal.pone.0127277 (PMC4436225; doi:10.1371/journal.pone.0127277)
Supplement: S2 Table — (DOCX) [file pone.0127277.s004.docx]

Table S2: Land cover classes frequencies (%) of the total city area of the main Italian cities studied.

| GlobCover Land Cover classes and descriptions | | Bari | Bologna | Catania | Florence | Genoa | Milan | Naples | Padua | Palermo | Rome | Turin |
| --- | --- | --- | --- | --- | --- | --- | --- | --- | --- | --- | --- | --- |
| C11 | Post-flooding or irrigated croplands (or aquatic) | 0.0 | 0.0 | 0.0 | 0.0 | 0.0 | 2.9 | 3.2 | 0.0 | 0.0 | 0.0 | 0.0 |
| C14 | Rainfed croplands | 5.9 | 30.4 | 16.1 | 7.6 | 0.3 | 22.1 | 12.3 | 38.4 | 16.7 | 25.7 | 9.3 |
| C20 | Mosaic cropland (50-70%) / vegetation (grassland / shrubland / forest) (20-50%) | 22.4 | 27.8 | 29.0 | 22.9 | 5.0 | 24.0 | 22.6 | 25.5 | 33.7 | 26.4 | 25.6 |
| C50 | Closed (>40%) broadleaved deciduous forest (>5m) | 0.8 | 16.5 | 3.3 | 30.0 | 72.0 | 13.3 | 15.2 | 6.8 | 0.8 | 13.8 | 26.9 |
| C70 | Closed (>40%) needleleaved evergreen forest (>5m) | 0.5 | 0.1 | 4.5 | 1.1 | 7.7 | 0.5 | 0.6 | 0.4 | 0.8 | 0.3 | 2.6 |
| C90 | Open (15-40%) needleleaved deciduous or evergreen forest (>5m) | 0.0 | 0.0 | 0.5 | 0.3 | 6.1 | 0.5 | 0.2 | 0.2 | 0.0 | 0.0 | 1.0 |
| C100 | Closed to open (>15%) mixed broadleaved and needleleaved forest (>5m) | 1.0 | 0.0 | 0.7 | 0.0 | 0.0 | 0.1 | 0.3 | 0.0 | 0.5 | 0.1 | 0.4 |
| C110 | Mosaic forest or shrubland (50-70%) / grassland (20-50%) | 0.0 | 0.0 | 1.3 | 0.0 | 0.0 | 0.0 | 0.0 | 0.0 | 0.0 | 0.0 | 0.1 |
| C120 | Mosaic grassland (50-70%) / forest or shrubland (20-50%) | 18.7 | 1.4 | 9.9 | 0.6 | 1.1 | 2.8 | 4.1 | 0.6 | 8.1 | 1.6 | 2.3 |
| C130 | Closed to open (>15%) (broadleaved or needleleaved, evergreen or deciduous) shrubland (<5m) | 43.1 | 15.4 | 24.5 | 26.8 | 3.9 | 7.4 | 21.1 | 14.8 | 24.0 | 20.4 | 14.0 |
| C150 | Sparse (<15%) vegetation | 0.0 | 0.0 | 0.0 | 0.0 | 0.0 | 0.0 | 0.0 | 0.0 | 0.0 | 0.0 | 1.2 |
| C190 | Artificial surfaces and associated areas (Urban areas >50%) | 7.6 | 8.4 | 10.2 | 10.7 | 3.9 | 26.4 | 20.4 | 13.3 | 15.4 | 11.7 | 16.6 |
